# Dataset for comparable evaluation of machine translation between 11 South African languages

**DOI:** 10.1016/j.dib.2020.105146

**Published:** 2020-01-14

**Authors:** Cindy A. McKellar, Martin J. Puttkammer

**Affiliations:** Centre for Text Technology, North-West University, South Africa

**Keywords:** Machine translation, Automatic evaluation, Natural language processing, Human language technology

## Abstract

This data article describes the Autshumato machine translation evaluation set. The evaluation set contains data that can be used to evaluate machine translation systems between any of the 11 official South African languages. The dataset is parallel with four reference translations available for each of the following languages: Afrikaans, English, isiNdebele, isiXhosa, isiZulu, Sepedi, Sesotho, Setswana, Siswati, Tshivenḓa and Xitsonga.

**Table:** Specifications Table

Subject	Computer Science
Specific subject area	Machine Translation which is an subfield of natural language processing/human language technology
Type of data	Text
How data were acquired	Professional human translation of text files.
Data format	Raw, Aligned
Parameters for data collection	Text in English from official governmental publications were considered for inclusion.
Description of data collection	English documents in pdf and Microsoft Word format were collected and converted to text format before translation.
Data source location	Institution: Centre for Text Technology, North-West University City/Town/Region: Potchefstroom Country: South Africa
Data accessibility	Repository name: SADiLaR Data identification number: Handle number: 20.500.12185/506 Direct URL to data: https://hdl.handle.net/20.500.12185/506

**Table:** 

**Value of the Data** • This parallel dataset can be used for the comparative evaluation of machine translation quality between any two of the 11 South African languages. • Any researcher working in the field of machine translation for the South African languages can benefit from this data. • Use of a single aligned evaluation set will enable accurate comparison of any machine translation systems developed for the South African languages in the future. • The data can also be annotated and used for the cross-language evaluation of other natural language processing core technologies and applications.

## 1 Data

The dataset contains 44 files (4 files for each of the 11 official languages of South Africa), each containing translations for 500 source sentences, created by a different professional human translator. All files are UTF8 encoded text files and contain one sentence per line. The 14 documents of origin are identified with mark-up in the files. As different automatic evaluation metrics require different file formats and mark-up, the files are distributed with no additional mark-up. Researchers can make the needed changes based on their specific system and evaluation requirements. The dataset is distributed under the Creative Commons Attribution 4.0 International license.^1^

Table 1 contains both the total (token) and unique word (type) counts of the four translations for each of the languages. There is also a column that contains the total word counts of all four reference translations combined. The final column contains an average type-token ratio for each language. The average type-token ratios were computed by first computing the type-token ratio for each of the four translations individually and then computing the average of the four ratios.

**Table 1:** Word count breakdown per language and translation.

	Translation 1		Translation 2		Translation 3		Translation 4		Totals		Avg. TTR
	Tokens	Types	Tokens	Types	Tokens	Types	Tokens	Types	Tokens	Types	
Afrikaans	9305	2296	9237	2332	9213	2315	9123	2375	36 878	3270	25.27%
English	9286	2223	9560	2104	9910	2080	9287	2317	38 043	3393	22.97%
isiNdebele	6524	3795	6285	3722	6716	3668	6397	3703	25 922	9215	57.47%
isiXhosa	7059	4203	6662	4035	6597	3893	6925	3969	27 243	10 466	59.11%
isiZulu	6666	3800	6841	3810	7017	3787	6528	3935	27 052	9568	56.74%
Sepedi	12 438	1973	11 539	2075	10 714	1854	11 642	2084	46 333	3695	17.26%
Sesotho	11 607	1892	11 394	2026	11 370	2018	10 497	1869	44 868	3656	17.41%
Setswana	11 531	2102	11 419	2043	12 826	2080	10 552	2055	46 328	4386	17.95%
Siswati	6117	3731	6292	3655	6624	3750	6658	3900	25 691	9470	58.57%
Tshivenḓa	11 241	1842	10 898	1819	11 283	1751	11 138	1746	44 560	2955	16.07%
Xitsonga	10 741	1984	11 094	1963	10 919	2069	10 996	1922	43 750	3912	18.15%

## 2 Experimental design, materials, and methods

Automatic evaluation of machine translation systems with metrics such as BLEU [1], NIST [2], TER [3], METEOR [4], etc. is an industry standard in response to the time-consuming and expensive nature of human evaluation. This evaluation set was specifically developed to enable comparative evaluation of machine translation systems [5],^2^ for the 11 official South African languages. The 11 official South African languages can generally be categorized into five language family groups. The conjunctively written Nguni languages include isiZulu, isiXhosa, isiNdebele and Siswati. The disjunctively written languages include the Sotho languages Sesotho, Setswana, Sepedi and Tshivenḓa and the disjunctively written Tswa-Ronga language Xitsonga. Finally, there are two Germanic languages, English and Afrikaans.

The evaluation set was created by randomly selecting documents from a collection of English documents originating from the South African government domain websites (*.gov.za). Clean-up to remove sections of the documents containing only contact information, repetition of whole sentences, lists and headings was performed. The source dataset contains 500 sentences from 14 documents on different topics from the government domain, and included speeches, press releases, health information and other information about government departments and services.

The 500 sentences were sent to 40 different professional translators (i.e. four translators per language) to translate, creating an evaluation set with four independent reference translations per language (except for English). In order to create the additional three English translations, one of the four translations from three of the languages was randomly selected and sent to three additional translators to translate back into English thereby completing the set of four translations for English.

The translators were each given strict instructions to translate the text using the latest orthographic and spelling rules for their language. Other limits were also placed on the translation to ensure that the resulting text will be suitable for use in machine translation evaluation. These limits were as follows:

• Each single sentence must be translated with a single sentence. Sentences may not be combined or divided.
• No information present in the source may be left out of the translation.
• No information may be added to the translation.
• No changes may be made to the sentence structure. This includes keeping dates, numbers and sentence types in the same format as they were in the source.

All translations where quality controlled to ensure that the translators followed these stipulations and that the resulting reference translations were as accurate as possible.

## Footnotes

1 CC BY 4.0 - https://creativecommons.org/licenses/by/4.0/.

2 https://mt.nwu.ac.za/.
